# Adaptation to Chronic-Cycling Hypoxia Renders Cancer Cells Resistant to MTH1-Inhibitor Treatment Which Can Be Counteracted by Glutathione Depletion

**DOI:** 10.3390/cells10113040

**Published:** 2021-11-05

**Authors:** Christine Hansel, Julian Hlouschek, Kexu Xiang, Margarita Melnikova, Juergen Thomale, Thomas Helleday, Verena Jendrossek, Johann Matschke

**Affiliations:** 1 Institute of Cell Biology (Cancer Research), University Hospital Essen, University of Duisburg-Essen, 45147 Essen, Germany; Christine.Hansel@uk-essen.de (C.H.); julian.hlouschek@stud.uni-due.de (J.H.); kexu.xiang@uk-essen.de (K.X.); margarita.melnikova@uk-essen.de (M.M.); Juergen.Thomale@uk-essen.de (J.T.); verena.jendrossek@uni-due.de (V.J.); 2 Science for Life Laboratory, Karolinska Institutet, 17121 Stockholm, Sweden; thomas.helleday@scilifelab.se

**Keywords:** chronic cycling hypoxia, radiation resistance, metabolic reprogramming, antioxidant capacity, redox homeostasis, ionizing radiation, TH1579, Karonudib, piperlongumine

## Abstract

Tumor hypoxia and hypoxic adaptation of cancer cells represent major barriers to successful cancer treatment. We revealed that improved antioxidant capacity contributes to increased radioresistance of cancer cells with tolerance to chronic-cycling severe hypoxia/reoxygenation stress. We hypothesized, that the improved tolerance to oxidative stress will increase the ability of cancer cells to cope with ROS-induced damage to free deoxy-nucleotides (dNTPs) required for DNA replication and may thus contribute to acquired resistance of cancer cells in advanced tumors to antineoplastic agents inhibiting the nucleotide-sanitizing enzyme MutT Homologue-1 (MTH1), ionizing radiation (IR) or both. Therefore, we aimed to explore potential differences in the sensitivity of cancer cells exposed to acute and chronic-cycling hypoxia/reoxygenation stress to the clinically relevant MTH1-inhibitor TH1579 (Karonudib) and to test whether a multi-targeting approach combining the glutathione withdrawer piperlongumine (PLN) and TH1579 may be suited to increase cancer cell sensitivity to TH1579 alone and in combination with IR. Combination of TH1579 treatment with radiotherapy (RT) led to radiosensitization but was not able to counteract increased radioresistance induced by adaptation to chronic-cycling hypoxia/reoxygenation stress. Disruption of redox homeostasis using PLN sensitized anoxia-tolerant cancer cells to MTH1 inhibition by TH1579 under both normoxic and acute hypoxic treatment conditions. Thus, we uncover a glutathione-driven compensatory resistance mechanism towards MTH1-inhibition in form of increased antioxidant capacity as a consequence of microenvironmental or therapeutic stress.

## 1. Introduction

Acquired therapy-resistance of solid tumors remains a major obstacle for successful cancer cure. The adverse tumor microenvironment (TME) represents an important factor for the development of resistance [1], in line with adaptation processes on single cancer cell level that drive malignant progression [2]. Facing the global increase of cancer incidence, it becomes even more important to uncover, characterize and subsequently target resistance mechanisms to established therapies [3].

In the context of therapy resistance mediated by an adverse TME, reduced availability of oxygen (tumor hypoxia) represents an important driver of genomic instability, malignant progression, and development of resistance to conventional therapies, such as radiotherapy [4]. Adaptation of cancer cells to a chronically hypoxic microenvironment involves increased flexibility to maintain the cellular redox homeostasis and an enhanced antioxidant capacity as a consequence of metabolic reprogramming; this helps cancer cells to cope with oxidative stress induced by increased cellular levels of reactive oxygen species (ROS) [5].

We and others described previously that increased antioxidant capacity is a particular feature of cancer cells with tolerance to cycling severe hypoxia and intermittent reoxygenation (subsequently termed “chronic-cycling hypoxia”) [5,6,7]. Independent of the selection procedure, improved antioxidant capacity involved increased levels of glutathione (GSH), a major cellular antioxidant molecule. We also demonstrated that the associated increased resistance to treatment with ionizing radiation (IR) could be overcome amongst others by inhibition of glutathione metabolism [6] or by inhibition of antioxidant-associated mitochondrial transport systems, leading to impaired cellular and mitochondrial redox homeostasis [8,9]. Importantly, work from other groups corroborated that targeting metabolic reprogramming associated with increased GSH levels is a promising concept for radiosensitization of cancer cells [10,11].

Increased levels of ROS occur during malignant progression, forcing cancer cells to functionally improve their antioxidant capacity [5,12]. But exposure to common therapeutic strategies, namely radiotherapy or several chemotherapeutic agents also triggers increased ROS formation and this contributes to their cytotoxic action and cell death [13,14]. Excessive accumulation of ROS induces oxidative damage to important cellular macromolecules, such as proteins, lipids, nucleotides, and the DNA. For example, oxidative DNA damage evokes activity of base excision repair (BER) to ensure cell survival [15,16]. Hence, targeting of BER, or targeting of the DNA damage response (DDR) or other DNA repair pathways are promising concepts for anticancer therapies [17], particularly when synthetic lethality with conventional treatment modalities like radiotherapy (RT) can be achieved [18,19].

Herein, the nudix family member enzyme MutT homologue-1 (MTH1) emerged as a promising new druggable target to impair the removal of oxidative DNA damage by BER; MTH1 hydrolyses the oxidized nucleotides 8-oxo-dGTP and 2-hydroxy-dATP and thus prevents the incorporation of damaged nucleotides into DNA [20,21].

To mimic potential therapeutic approaches of MTH1-inhibition, we used the clinically relevant compound TH1579 (Karonudib). Of note, this small molecule inhibitor is currently evaluated in phase I clinical trials (NCT03036228, NCT04077307).

Even though there is broad evidence for the efficacy of MTH1-inhibition in cancer cells under preclinical settings, it has been reported that cancer cells develop resistance against MTH1. For example, an MTH1-independent 8-oxo-dGTPase in human cancer cells can drive resistance to MTH1-inhibitors due to functional redundancy [22]. In contrast, redox imbalances or increased hypoxia signaling may sensitize cancer cells to MTH1-inhibition, potentially by impairing functional orchestration of BER involving MTH1 [23]. In fact, DNA repair is severely altered under hypoxic conditions, e.g., by reduced expression of several BER factors, and this might increase the vulnerability of hypoxic cancer cells towards oxidative DNA damage [24,25].

So far, mechanisms of MTH1 resistance are not yet completely understood; moreover, valid stratification markers are missing, particularly for heterogeneous tumors with larger hypoxic cell fractions.

We hypothesized, that the improved tolerance to oxidative stress will increase the ability of the cancer cells to cope with ROS-induced damage to free deoxy-nucleotides (dNTPs) required for DNA repair and may thus contribute to acquired resistance of cancer cells in advanced tumors to antineoplastic agents inhibiting the nucleotide-sanitizing enzyme MTH1, ionizing radiation (IR) or both. Therefore, we aimed to explore potential differences in the sensitivity of cancer cells exposed to acute and chronic-cycling anoxia/reoxygenation stress (chronic-cycling hypoxia) to the clinically relevant MTH1-inhibitor TH1579 and to test whether a multi-targeting approach combining the glutathione withdrawer piperlongumine (PLN) and TH1579 may be suited to increase cancer cell sensitivity to TH1579 alone and in combination with IR.

## 2. Materials and Methods

### 2.1. Reagents, Cell Lines and Culturing Conditions

All chemicals were purchased from Sigma Aldrich (St. Louis, MO, USA) if not stated otherwise. NCI-H460 lung adenocarcinoma cells and T98G glioblastoma cells were obtained from ATCC (Bethesda, MD, USA). All cells were authenticated through STR sequencing and analysis performed with the GenePrint 10 System (Promega Corp., Madison, WI, USA) in accordance with the ANSI/ATCC ASN-0002-2011 standardization of STR profiling guidelines and routinely checked for mycoplasma. NCI-H460 or T98G cells with tolerance to cycling severe hypoxia/re-oxygenation stress (“anoxia-tolerant cells”) were generated by exposure to 16 cycles (T98G) or 25 cycles (NCI-H460) of severe hypoxia (48 h, <0.1% O_2_) and intermittent re-oxygenation (120 h air plus 5% CO_2_ referred as 20% O_2_) as described earlier in more detail [6,8,9,26]. Control cells (“oxic cells”) were cultured in parallel under standard ambient O_2_ conditions (20% O_2_ plus 5% CO_2_) [20]. Upon selection, cancer cells were routinely grown in RPMI 1640 medium. supplemented with 10% (*v*/*v*) fetal calf serum (Gibco/Life Technologies, Carlsbad, CA, USA) and maintained in a humidified incubator at 37 °C and 5% CO_2_ (referred to as “normoxia“ or “normoxic conditions“). For severely hypoxic conditions, cells were grown in a humidified hypoxia workstation (In vivo 400, Ruskinn Technology Ltd., Bridgend, UK and Whitley H35 Hypoxystation, Don Whitley Scientific, Bingley, West Yorkshire, UK) at 37 °C, 0.2% O_2_, and 5% CO_2_ (referred to as “hypoxia” or “hypoxic conditions“).

### 2.2. Irradiation and Treatment

Piperlongumine (PLN, [27]) was purchased from Cayman (Ann Arbor, MI, USA). TH1579 [28] was provided by Thomas Helleday (SciLife Lab, Karolinska Institutet, Stockholm, Sweden). Irradiation was performed as previously described [6,8,9]. In brief, cells were irradiated at room temperature with an X-ray machine (Precision X-ray Inc., North Branford, CT, USA) operated at 320 kV, 12.5 mA with a 1.65 mm Al filter, at a distance of 50 cm and a dose rate of 3.71 Gy/min. Cells were returned to the incubator immediately after exposure to 5–10 Gy IR. For irradiation under hypoxic conditions, cells were subjected to 2 h of pre-incubation in hypoxia to allow adaptation. Then, hypoxic cell dishes were kept in BD GasPak EZ Pouch System (Becton Dickinson, Heidelberg, Germany). For combined multimodal treatments, PLN and/or TH1579 were added 2 h prior to irradiation.

### 2.3. qRT-PCR Analysis

qRT-PCR analysis was performed as previously described [6,8,9]. In brief, RNA isolation was performed with the Qiagen RNeasy Minikit (Qiagen, Hilden, Germany) according to the manufacturer’s protocol. cDNA was synthesized from 1 μg of total RNA using QuantiTect Reverse Transcription Kit (Qiagen, Hilden, Germany). Specific primers were synthesized based on available sequences for each mentioned gene. Primer sets were designed with the Blast web tool (U.S. National Centre for Biotechnology Information, Bethesda, MD, USA). PCR products were 150–200 bp in size. We used published β2-microglobulin (B2M) primer sequences as housekeeping gene [29]. qRT–PCR and cycling conditions were performed using specific oligonucleotide primers (B2M forward: TGCTGTCTCCATGTTTGATGTATCT; reverse: TCTCTGCTCCCCACCTCTAAGT; MTH1 forward: TTCTGCACAGACAGCATCCA; reverse: ATGGTGTCCTGACCCTGGAA; FEN1 forward: TGACTGCCAGTGAAGCCAAA; reverse: CTCCTCGATGCTCTTGTGCT; OGG1 forward: CCCCCAGACCAACAAGGAAC; reverse: TTGTGAATCCCCTCTCCCGA) and by using qPCR kit for SYBR^®^ Green I, 6-Carboxyl-X-Rhodamine (ROX) (Eurogentec, Cologne, Germany) according to the manufacturer’s protocol. Reactions were carried out on an ABI Prism 7900HT using MicroAmp Optical 384 well Reaction plates (Applied Biosystems by Life Technologies, Bleijswijk, The Netherlands) and BIO-RAD PCR Sealers Microseal “B” Film Adhesive seal (optically clear; Bio-Rad, Munich, Germany). Melting curves were obtained after each PCR run and showed single PCR products. cDNAs were run in triplicate, without reverse transcriptase and no-template controls were run in duplicates. Expression ratios were calculated using the geometric mean expression of the housekeeping gene B2M to normalize the expression data for the genes of interest according to the 2-ΔΔCt-method as described by others [30].

### 2.4. Assessment of Oxidative DNA Damage

To quantify the amount of oxidative DNA damage, cells were stained for 8-hydroxy-guanosine (8-oxo-G) and analyzed by fluorescence microscopy as previously described [31]. In more detail, cells were plated, incubated overnight, and exposed for 24 h to the respective treatment. Subsequently, cells were washed, harvested and spotted on Superfrost Plus Gold slides (Thermo Fisher Scientific, Waltham, MA, USA). Treatment for 15 min with 1 mM H_2_O_2_ was used as positive control. After drying of the spotted cell suspensions at room temperature, cells were fixed in methanol, placed in alkali-solution (70 mM NaOH, 140 mM NaCl, 40% methanol) and washed. Pepsin and proteinase K were added in separate steps and incubated for 10 min each at 37 °C. Slides were then blocked in skim-milk solution (5%) for 30 min. Then, the primary mouse anti-8-hydroxyguanosine antibody (Abcam, Cambridge, UK) in BSA-solution (1:20,000) was added for an overnight incubation at 4 °C. The secondary antibody Cy-3 conjugated AffiniPure Anti-Mouse IgG (Abcam, Cambridge, UK) was added at a concentration of 0.05 μg/mL and incubated for 1 h at 37 °C. Nuclear staining was performed using DAPI (Thermo Fisher Scientific). Afterwards, cover slips were mounted using Immuno Select Antifading Mounting Medium (Squarix Biotechnology, Marl, Germany). Fluorescence microscopic pictures were taken with a Zeiss Cell Observer.Z1 fluorescent microscope with ApoTome using the Zeiss Zen software (Carl Zeiss, Goettingen, Germany). Quantitative analysis was performed with a Zeiss Axioplan fluorescence microscope and the Ahrens ACAS imaging system (Ahrens ACAS, Hamburg, Germany). Fluorescence signals of Cy3 were measured in the nuclear area of individual cells, normalized to the respective DNA content (integrated DAPI signals) and expressed as arbitrary fluorescence units (AFU) representing the relative levels of 8-oxo-G. 50 to 100 cells were analyzed for each treatment and cell line condition.

### 2.5. Determination of GSH Levels

GSH/GSSG ratios were measured using GSH/GSSG Glo Assay Kit (Promega, Madison, WI, USA) according to manufacturer´s protocol. Briefly, adherent cells in 96 well plates were lysed and: (i) GSSG was reduced to GSH or (ii) GSH was blocked to measure total GSH or GSSG individually using a luciferase-coupled enzymatic reaction.

Levels of reduced glutathione (GSH) were determined by using monochlorobimane (MCB, Thermo Scientific, Waltham, MA, USA) as described previously [9] using 10 μM MCB with 15 min of pre-incubation. To rule out alterations in the speed of dye metabolism instead of alterations in absolute cellular GSH levels, kinetic measurements of fluorescence were performed for 15 min at 37 °C. Each assay contained cells treated with H_2_O_2_ to deplete GSH as a negative control. In case of 24 h treatment cells were subsequently fixed with 4% paraformaldehyde and stained with 10 μg/mL solution of Hoechst 33342 (Thermo Scientific, Waltham, MA, USA) for normalization to cell number. Assay was conducted in 96-well plates using a BioTek Synergy Microplate reader for fluorescence measurements (BioTek, Winooski, NH, USA).

### 2.6. Flow Cytometry Analysis

The fraction of dead cells was quantified by flow cytometry (FACS Calibur, Becton Dickinson, Heidelberg, Germany; FL-2) of PI–stained cells as described previously [6,8,9]. Cells were incubated for 30 min in the dark with PI (10 μg/mL, Thermo Scientific, Waltham, MA, USA) in PBS and measured within 1 h.

To quantify cellular ROS production, cells were stained for 30 min at 37 °C with 5 μM Dihydroethidium (DHE) in RPMI 1640 medium (Molecular Probes/Invitrogen, Carlsbad, CA, USA). DHE-positive cells were detected by flow cytometry (BD FACS Calibur, Becton Dickinson; FL-2). ROS-production was evaluated by quantification of the fraction of DHE-positive cells (at least 10,000) with higher fluorescence.

### 2.7. Colony Formation Assays

The influence of the respective treatments on long-term survival was analyzed by comparing the clonogenic survival of treated and untreated cells cultured either under normoxic or severely hypoxic conditions as described previously [6,8,9]. In brief, for treatment in normoxia, exponentially growing cells were seeded in tissue culture flasks, incubated under standard culturing conditions (20% O_2_, 5% CO_2_, 37 °C) and irradiated 24 h later (0 or 5 Gy) without or with prior treatment with PLN (10 μM) and/or TH1579 (1 μM), respectively. PLN and/or TH1579 treatment was performed 2 h prior to irradiation. For treatment in hypoxia, tissue culture flasks of exponentially growing cells were exposed to severe hypoxia (0.2% O_2_) 2 h prior to PLN and/or TH1579 treatment and 4 h prior to irradiation, respectively. After completion of the treatments, cells were incubated for 24 h under normoxic or hypoxic conditions, then washed, collected (0.05% Trypsin, 0.01 EDTA), and plated to 6 well plates at densities of 200 to 3200 cells per well in inhibitor-free media (delayed plating). Cell viability was checked before plating the cells by using CASY COUNT (Omni Life Science, Bremen, Germany) and only the number of viable cells was used for the calculation of necessary cell numbers for plating. Plates were subsequently incubated for 9 days under standard normoxic conditions before quantification of colony formation. For that, cells were fixed in 3.7% formaldehyde and 70% ethanol, stained with 0.05% Coomassie blue, and colonies of at least 50 cells were counted by GelCount (Oxford Optronix, Oxfordshire, UK). The plating efficiency and surviving fraction (SF) to corresponding normoxic and hypoxic controls were calculated as described elsewhere [32].

### 2.8. Statistics

Data represent mean values of at least 3 independent experiments ± standard error of mean (SEM). Data analysis was performed by two-way ANOVA tests using parametric methods and employing Tukey multiple comparison post-test where appropriate or by unpaired Student’s *t*-test with Holm-Sidak correction for multiple comparisons using Prism 6 software (Graph Pad Inc., San Diego, CA, USA). If not indicated differently, asterisks above bars refer to comparison with corresponding controls. *p*-values ≤ 0.05 were considered significant.

## 3. Results

### 3.1. Inhibition of Glutathione System Overcomes Acute and Chronic-Cycling Hypoxia-Induced Reduction of Cell Death upon MTH1-Inhibition in Combination with IR

**Figure 1:**
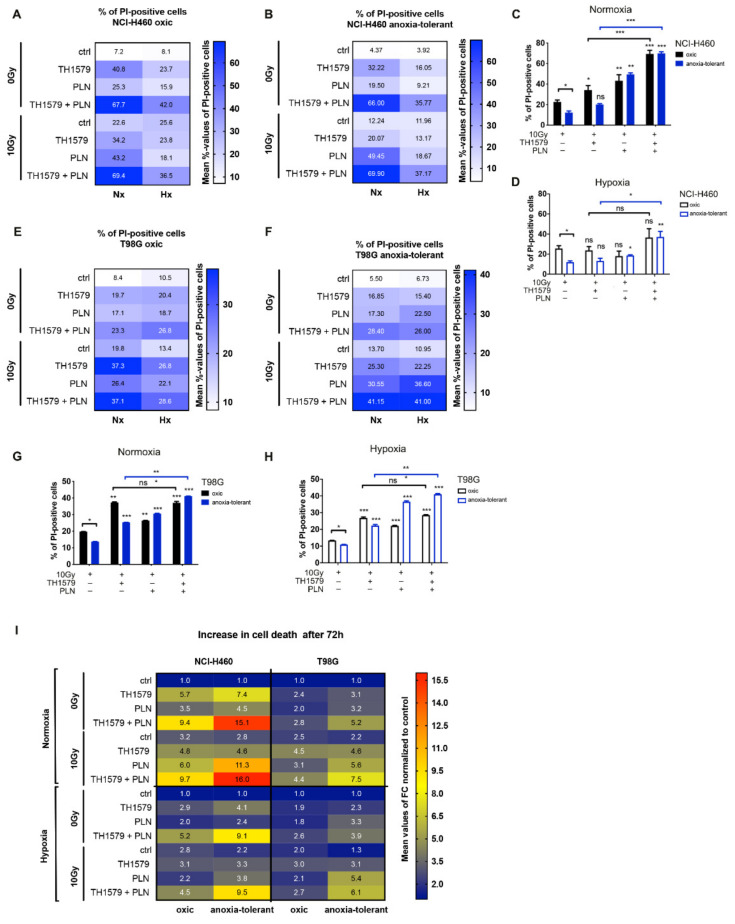
Inhibition of MTH1 combined with inhibition of glutathione regeneration overcomes cell death resistance induced by adaptation to chronic-cycling hypoxia. Impact of MTH1 inhibition by TH1579 (1 μM) alone and in combination with PLN (10 μM) and exposure to ionizing radiation (IR, 10 Gy) on cell death under normoxic (Nx) and hypoxic (Hx) conditions was examined. (**A**,**B**) Heatmap representing mean % of measured Propidium iodide (PI)-positive NCI-H460 oxic control (**A**) and anoxia-tolerant (**B**) cell lines under Nx and Hx. (**C**,**D**) Fraction of dead (PI-positive) NCI-H460 cells 72 h after respective inhibitor treatments with 10 Gy IR under Nx (**C**) and Hx (**D**) determined by flow cytometry. (**E**,**F**) Heatmap representing mean % of PI-positive T98G oxic control (**E**) and anoxia-tolerant (**F**) cell lines under Nx and Hx. (**G**,**H**) Fraction of PI-positive T98G cells 72 h after respective inhibitor treatments with 10 Gy IR under Nx (**G**) and Hx (**H**). (**I**) Representation of the treatment-induced increase in cell death in anoxia-tolerant and respective oxic control NCI-H460 and T98G cancer cells under normoxia and hypoxia. Cell death levels were normalized to levels of untreated controls and represented as fold changes (FC) in a heat map. Measured mean % of PI-positive cells used to calculate FC can be found in Supplementary Materials Figure S1E. Mean values ± SEM are shown, *n* = 3–5 (ns: not significant, * *p* ≤ 0.05, ** *p* ≤ 0.01, *** *p* ≤ 0.001; *t*-test or 2-way ANOVA with Tukey post-test where applicable).

Our previous work revealed increased radiosensitivity of chronic cycling hypoxia-selected anoxia-tolerant cancer cells [6,26], which was linked to improved antioxidant capacity of anoxia-tolerant cancer cells [6,8,9]. We hypothesized that improved antioxidant defense of cancer cells exposed to chronic-cycling hypoxia might limit the efficacy of MTH1-inhibitors, particularly in combination with IR. Thus, we first examined the levels of radiation-induced early cell death of oxic and anoxia-tolerant NCI-H460 lung and T98G glioblastoma cancer cells upon inhibition of MTH1 by TH1579 or treatment with the glutathione-antagonist piperlongumine (PLN) alone (Figure 1A,B,E,F and Figure S1) and in multimodal combination treatments with a single high dose of IR (10 Gy) after 72 h.

Chronic-cycling hypoxia reduced basal and radiation-induced cell death levels of anoxia-tolerant NCI-H460 and T98G cancer cells (Figure 1A–H and Figure S1) in agreement with our own previous results [6]. TH1579 treatment alone induced significant cell death levels in oxic and anoxia-tolerant NCI-H460 and T98G cell lines under normoxic (20.9% O_2_, Figure 1A–F and Figure S1) and lead to reduced cell death levels under hypoxic (0.2% O_2_) conditions (Figure 1A–F and Figure S1). Interestingly, combining TH1579 with PLN potentiated cell death levels in tested cell lines (Figure 1A–H and Figure S1). However, combination of PLN with a single high dose irradiation of 10 Gy increased cell death levels of oxic and anoxia-tolerant NCI-H460 and T98G cell lines to a greater extent compared to PLN-treatment alone (Figure 1A–H and Figure S1E). Here, the treatment-induced PLN effect on short-term cell death induction in combination with IR was more pronounced in anoxia-tolerant cancer cells (Figure 1I). Again, acute hypoxia reduced the cytotoxicity of used combination treatments (Figure 1A–H).

Inhibition of MTH1 alone did not induce higher cell death levels in anoxia-tolerant NCI-H460 cells by a single high dose irradiation with 10 Gy under normoxic (Figure 1B,C and Figure S1E) and acute hypoxic (0.2% O_2_) conditions (Figure 1B,D and Figure S1E). In contrast, anoxia-tolerant T98G glioblastoma cancer cells experienced increased cell death levels upon combination treatment by using TH1579 and IR under normoxic (Figure 1F,G and Figure S1E) and hypoxic (Figure 1F,H and Figure S1E) conditions. Multimodal treatment by using TH1579 and PLN increased levels of radiation-induced cell death in anoxia-tolerant NCI-H460 and T98G cells to the levels of oxic controls, thereby counteracting reduced cell death levels of anoxia-tolerant cancer cells induced by adaptation to chronic cycling hypoxia, and possibly overcoming increased radioresistance (Figure 1C,D,G,H and Figure S1E). Remarkably, calculating treatment-induced fold changes of increase in cell death revealed, that combined treatment of TH1579 and PLN potentiated radiation-induced cell death (Figure 1I) with more pronounced effects in anoxia-tolerant cancer cells. The observation of increased cell death induction induced by multimodal treatment in combination with IR in anoxia-tolerant cells corroborate our hypothesis of reduced efficacy of MTH1-inhibitors in case of increased antioxidant defense observed in case of adaptation to chronic-cycling hypoxia as reported previously [6,8,9]. In conclusion, the described specific vulnerability of anoxia-tolerant cancer cells could be exploited to counteract developed radioresistance in short-term survival of cancer cells derived from different entities. However, the combination treatment of TH1579 and PLN for 72 h revealed high cytotoxicity on the tested cell lines without IR-treatment.

### 3.2. Ionizing Radiation Increases Expression of MTH1 in Normoxia While Acute and Chronic Cycling Hypoxia Decreases Their Expression

**Figure 2:**
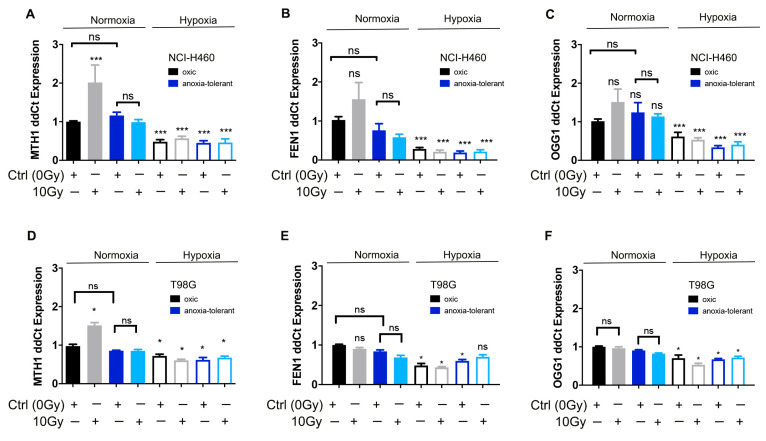
Ionizing radiation (IR) increases expression of *MTH1* and BER genes in normoxia while acute hypoxia decreases their expression. Basal expression of *MTH1*, *OGG1* and *FEN1* was determined in cancer cells with tolerance to chronic-cycling hypoxia (anoxia-tolerant) and respective oxic control cells 24 h after exposure to severe hypoxia and/or 10 Gy of IR. (**A**) qRT-PCR analysis of *MTH1* expression normalized to oxic control in NCI-H460 cells. (**B**) qRT-PCR analysis of *FEN1* expression normalized to oxic control in NCI-H460 cells. (**C**) qRT-PCR analysis of *OGG1* expression normalized to oxic control in NCI-H460 cells. (**D**) qRT-PCR analysis of *MTH1* expression normalized to oxic control in T98G cells. (**E**) qRT-PCR analysis of *FEN1* expression normalized to oxic control in T98G cells. (**F**) qRT-PCR analysis of *OGG1* expression normalized to oxic control in T98G cells. Mean values ± SEM are shown, *n* = 3 (ns: not significant, * *p* ≤ 0.05, *** *p* ≤ 0.001; *t*-test).

Next, we aimed to gain insight into the reduced efficacy of MTH1-inhibitor (TH1579) induced by acute and chronic-cycling hypoxia. Therefore, we examined a potential deregulation of *MTH1* expression by microenvironmental or therapeutic stress by comparing the mRNA expression of *MTH1* in cells cultured in standard culturing conditions, cells exposed to acute (0.2% O_2_, 24 h) or chronic-cycling hypoxia (anoxia-tolerant cells) or IR-treatment (10 Gy, 24 h) respectively. Exposure to acute hypoxia decreased expression of *MTH1* in NCI-H460 lung (Figure 2A) and T98G glioblastoma (Figure 2D) anoxia-tolerant and oxic control cancer cells. In contrast, single high dose irradiation with 10 Gy increased expression of *MTH1* in oxic control cells under normoxic conditions, but not in anoxia-tolerant cells (Figure 2A,D). Instead, irradiation under acute hypoxic conditions did not induce *MTH1* expression, neither in oxic control nor in anoxia-tolerant NCI-H460 cells (Figure 2A,D). Moreover, to explore an associated deregulation of BER, we additionally analyzed gene expression of two additional BER proteins, namely 8-oxoguanine glycosylase (*OGG1*) and Flap endonuclease 1 (*FEN1*) under the above conditions by using qRT-PCR [15,16].

While exposure to acute hypoxia decreased expression of BER genes *MTH1*, *OGG1* and *FEN1*, we did not observe differences in the expression of these genes between anoxia-tolerant and oxic control NCI-H460 lung (Figure 2B,C) or T98G glioblastoma (Figure 2E,F) cancer cells in acute hypoxic conditions.

These observations corroborate previous observations of reduced BER in acute hypoxia [28] and additionally hint to a decreased reliance of irradiated anoxia-tolerant cancer cells on MTH1 due to increased antioxidant capacity.

### 3.3. Exposure to IR or Acute Hypoxia Triggers Antioxidant Defense Offering Opportunity for Multimodal Inhibition of MTH1 and Glutathione Regeneration

We hypothesized that cancer cells with high intracellular GSH levels might be less dependent on MTH1-induced detoxification of oxidized nucleotides. Therefore, we analyzed potential differences in the ability of anoxia-tolerant and oxic control cells to enhance cellular GSH levels in response to ROS-inducing irradiation. Moreover, we aimed to explore if and how exposure to acute hypoxia would differentially alter IR-induced changes in cellular GSH levels in anoxia-tolerant cells and their oxic controls.

**Figure 3:**
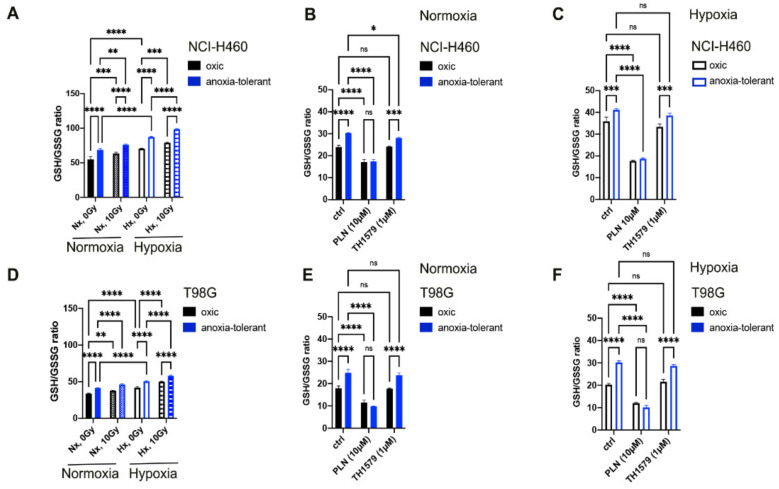

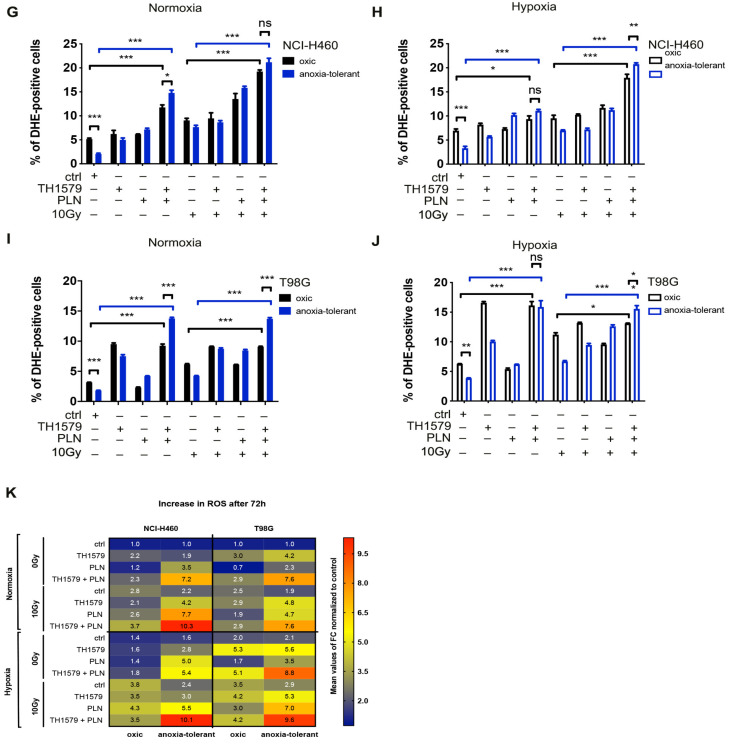
Exposure to IR or acute hypoxia triggers antioxidant defense which can be counteracted by inhibition of MTH1 and glutathione regeneration. Impact of IR and/or acute hypoxia on antioxidant capacity and ROS formation of oxic and anoxia-tolerant NCI-H460 and T98G cells and the potential of PLN and MTH1-inhibitor TH1579 for ROS modulation are shown. (**A**) GSH/GSSG ratios in NCI-H460 oxic and anoxia-tolerant cells 24 after exposure to 10 Gy IR, acute hypoxia or both. (**B**,**C**) GSH/GSSG ratios of NCI-H460 oxic and anoxia-tolerant cancer cells after 2 h of PLN (10 μM)—or TH1579 (1 μM) -treatment under normoxic (**B**) and hypoxic (**C**) conditions. (**D**) GSH/GSSG ratios in T98G oxic and anoxia-tolerant cells 24 h after exposure to 10 Gy IR, acute hypoxia or both. (**E**,**F**) GSH/GSSG ratios of T98G oxic and anoxia-tolerant cancer cells after 2 h of PLN—or TH1579 -treatment under normoxic (**E**) and hypoxic (**F**) conditions. (**G**,**H**) Fraction of DHE-positive cells stained for cellular ROS determined by flow cytometry in NCI-H460 oxic and anoxia-tolerant cells after 24 h of the respective treatments under normoxic (**G**) and severe hypoxic (**H**) conditions. (**I**,**J**) Fraction of DHE-positive cells stained for cellular ROS determined by flow cytometry in T98G oxic and anoxia-tolerant cells after 24 h of the respective treatments under normoxic (**I**) and severe hypoxic (**J**) conditions. (**K**) Representation of the treatment-induced increase in ROS in anoxia-tolerant and respective oxic control NCI-H460 and T98G cancer cells under normoxia and hypoxia. Measured ROS-levels were normalized to levels of untreated controls and represented as mean fold change (FC) in a heat map. Heat map indicating mean fold change of ROS induction in NCI-H460 or T98G oxic and anoxia-tolerant cells after 24 h of the respective treatments under normoxic and severe hypoxic conditions. Mean values ± SEM are shown, *n* = 3 (ns: not significant, * *p* ≤ 0.05, ** *p* ≤ 0.01, *** *p* ≤ 0.001, **** *p* ≤ 0.0001; 2-way ANOVA with Tukey post-test).

As already observed in our earlier work [6,8,9], NCI-H460 and T98G anoxia-tolerant cells were characterized by higher basal GSH levels compared to oxic control cells (Figure S2), but also displayed elevated GSH/GSSG ratios under the different treatment conditions analyzed in the present study (Figure 3A,D). Exposure to acute hypoxia for 24 h significantly increased GSH/GSSG ratios in both, oxic and anoxia-tolerant NCI-H460 (Figure 3A) and T98G cells (Figure 3D). While under normoxic conditions only a slight increase of GSH/GSSG ratios was observed 24 h after IR, we observed a significant increase in GSH/GSSG ratios 24 h after irradiation when treatment was performed under severely hypoxic conditions (Figure 3A,D).

Thus, exposure to IR resulted in a more pronounced increase in cellular GSH levels 24 h after irradiation (Figure S2) suggesting a reactive increase of GSH formation in response to aggravation of oxidative stress within 24 h after IR treatment.

Our previous work had also revealed that treatment with the documented GSH-depleting drug PLN [27,33] restored radiation-induced ROS production and cell death in anoxia-tolerant cells to the levels of oxic control cells, thereby overcoming adaptation-associated increased radioresistance [6]. Pretreatment for 2 h with PLN significantly decreased GSH/GSSG ratios under both normoxic (Figure 3B,E) and hypoxic (Figure 3C,F) conditions. Importantly, the relative reduction of GSH/GSSG ratios was always more pronounced in anoxia-tolerant cells compared to oxic control cells upon PLN treatment (Figure 3B,E), thereby enhancing our earlier findings with respect to direct evidence of PLN-induced GSH depletion in our experimental model. Furthermore, pretreatment with TH1579 did not have any effect on the GSH/GSSG ratios of both, oxic and anoxia-tolerant NCI-H460 (Figure 3B,C) and T98G (Figure 3E,F) cell lines. Next, we investigated the effect of MTH1-inhibition alone or in combination with PLN on cellular ROS in NCI-H460 lung cancer and T98G glioblastoma cells. While treatment with MTH1 inhibitor TH1579 alone did not significantly induce ROS formation after 24 h of treatment, combination of PLN and TH1579 led to increased ROS levels in NCI-H460 lung (Figure 3G,H) and T98G glioblastoma (Figure 3I,J) cancer cell lines. In more detail, combination treatment was associated with a significantly higher ROS-increase than PLN treatment alone, with even more pronounced effects on anoxia-tolerant cells compared to oxic control cells under both normoxic (Figure 3G,I) and hypoxic conditions (Figure 3H,J). These differences are even more clearly depicted when comparing fold change induction of ROS (Figure 3K).

Importantly, the observed effect on ROS-increase could be further extended when combining PLN, TH1579 and a single high-dose irradiation of 10 Gy (Figure 3K), hinting to a potentially induced vulnerability against IR. Taken together, or data demonstrate a compensatory mechanism of glutathione-induced TH1579 resistance triggered by acute and chronic-cycling hypoxia.

### 3.4. Radiation Triggers Oxidative DNA Damage and Synergizes with Multimodal Targeting of MTH1 and Glutathione System

**Figure 4:**
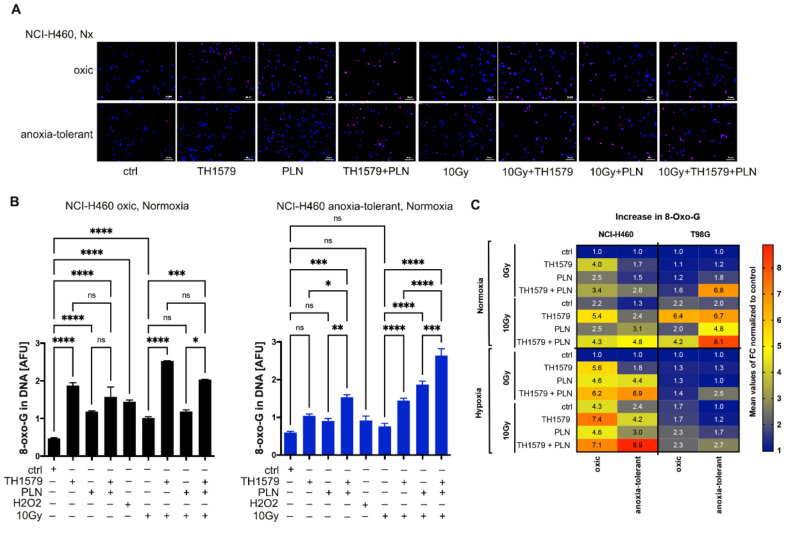
Inhibition of MTH1 alone and combined with inhibition of glutathione regeneration leads to increased oxidative DNA damage, especially in acute hypoxia. Effect of IR and/or acute hypoxia on formation of oxidative DNA damage in oxic and anoxia-tolerant NCI-H460 cells and the alterations upon 24 h of treatment with PLN and TH1579 were investigated. (**A**) Representative micrographs of 8-oxo-G formation in NCI-H460 oxic and anoxia-tolerant cells treated under normoxic conditions analyzed by fluorescence microscopy. Scale bars display 50 μm. (**B**) Bar graphs indicating arbitrary fluorescence units (AFU) of 8-oxo-G of oxic (left panel) and anoxia-tolerant (right panel) NCI-H460 cells in nucleus area (**C**) Heatmap indicating fold changes of 8-oxo-G formation in NCI-H460 or T98G oxic and anoxia-tolerant cells under different treatments compared to untreated controls under normoxic and severe hypoxic conditions. Bar graphs with measured AFU used to calculate FC can be found in Supplementary Materials Figure S3. Mean values ± SEM are shown, *n* = 3 (* *p* ≤ 0.05, ** *p* ≤ 0.01, *** *p* ≤ 0.001, **** *p* ≤ 0.0001; *t*-test).

Subsequently, we included the assessment of oxidative DNA damage into our analysis by staining for 8-oxo-G using fluorescence microscopy to further investigate if the observed alterations in ROS and GSH levels are also reflected by oxidative DNA damage. Treatment with TH1579 alone for 24 h led to increased oxidative DNA damage in NCI-H460 oxic control cells to a higher extent than in anoxia-tolerant cells (Figure 4A,B). Furthermore, significant increase in 8-oxo-G formation occurred in NCI-H460 and T98G anoxia-tolerant cells when combining TH1579 treatment and PLN, but not in oxic control cells (Figure 4B,C and Figure S2A). Again, this increase could be further extended by multimodal treatment combining PLN, TH1579 and a single high-dose irradiation of 10 Gy (Figure 3B,C and Figure S3), in line with our previous measurements of ROS levels. Interestingly, enhanced formation of oxidative DNA damage upon combination of TH1579 and PLN and/or additional IR-treatment also occurred in anoxia-tolerant T98G glioblastoma cells when compared to their oxic controls (Figure 4C and Figure S3B,C). Of note, oxidative DNA damage in NCI-H460 cells was more pronounced in acute hypoxia compared to normoxia, regardless of the performed treatments (Figure 4C and Figure S3C).

Hence, the observed resistance of anoxia-tolerant cancer cells towards MTH1-inhibition could be disrupted by targeting the antioxidant defense, leading to enhanced accumulation of ROS, GSH depletion and oxidative DNA damage. Even more important, the described specific vulnerability of anoxia-tolerant cancer cells could be exploited to counteract developed radioresistance.

### 3.5. Inhibition of MTH1 Combined with Inhibition of Glutathione Regeneration Overcomes Radioresistance Induced by Adaptation to Chronic-Cycling Hypoxia

**Figure 5:**
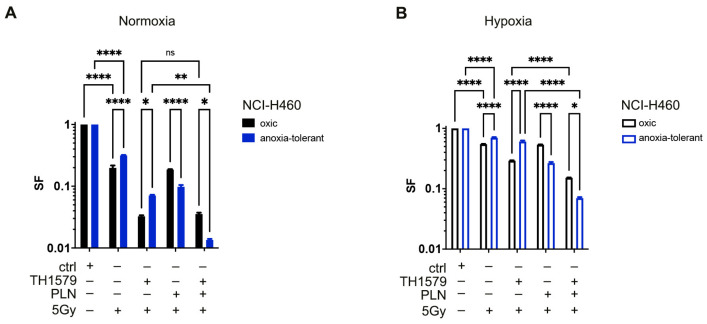
Inhibition of MTH1 combined with PLN overcomes radioresistance induced by adaptation to chronic-cycling hypoxia. Impact of MTH1 inhibition by TH1579 (1 μM) alone and in combination with GSH-Inhibitor PLN (10 μM) and exposure to IR (5 Gy) on clonogenic survival under normoxic and hypoxic conditions was examined. (**A**) Survival fraction (SF) of clonogenic survival of NCI-H460 oxic and anoxia-tolerant cells upon respective drug treatments and IR with 5 Gy under normoxic conditions is presented in a bar graph. (**B**) Survival fraction (SF) of clonogenic survival of NCI-H460 oxic and anoxia-tolerant cells upon respective drug treatments and IR with 5 Gy under hypoxic conditions is presented in a bar graph. Mean values ± SEM are shown, *n* = 3 (ns: not significant, * *p* ≤ 0.05, ** *p* ≤ 0.01, **** *p* ≤ 0.0001; 2-way ANOVA with Tukey post-test).

We hypothesized that cycling severe hypoxia/reoxygenation stress might be responsible for enhancing not only resistance of solid tumors to IR but also to MTH1-inhibition by TH1579. We thus speculated that we might be able to increase vulnerability to TH1579 or combined treatment of TH1579 and IR by targeting the cellular redox homeostasis, by using a glutathione withdrawer piperlongumine (PLN). Since the combination treatment of TH1579 and PLN for 72 h revealed high short-term cytotoxicity on the tested cell lines without IR-treatment (Figure 1) we used a previously described indirect plating method for the analysis of long-term clonogenic survival [6,8,9,26], thus reducing the drug-treatment period to 24 h and the radiation dose to 5 Gy. Therefore, we analyzed long-term clonogenic survival after irradiation with a dose of 5 Gy alone or in combination with TH1579, PLN or both under normoxic (Figure 5A and Figure S4A) and severe hypoxic conditions (Figure 5B and Figure S4B).

TH1579 treatment slightly increased sensitivity of oxic and anoxia-tolerant NCI-H460 cancer cells to IR but was not able to counteract increased radioresistance induced by chronic-cycling hypoxia (Figure 5A and Figure S4C,D). However, combining TH1579 with PLN efficiently decreased clonogenic survival of irradiated anoxia-tolerant NCI-H460 cells under normoxic conditions (Figure 5A and Figure S4D). Of note, this effect was more pronounced in irradiated anoxia-tolerant NCI-H460 cells than the oxic control cells. The same treatment schedule was also performed under acute hypoxic conditions (Figure 5B and Figure S4E,F). Despite the decreased efficacy of IR on clonogenic survival under hypoxic conditions, we could again observe an increased resistance of anoxia-tolerant cells to TH1579 in comparison to oxic controls (Figure 5B and Figure S4E,F). Importantly, combination of TH1579 and PLN again efficiently decreased clonogenic survival of anoxia-tolerant NCI-H460 cells and sensitized them to the cytotoxic action of IR under acute hypoxia (Figure 5B and Figure S4E,F).

In summary, the specific susceptibility of anoxia-tolerant cancer cells, namely enhanced antioxidant capacity, could be exploited to counteract evolved resistance against MTH1-inhibition alone and in combination with IR, affecting both short- and long-term survival of cancer cells from different entities. Furthermore, inhibition of MTH1 in combination with cellular antioxidant defense might be suited for targeting phenotypic heterogeneity of cancer cells caused by adaptation to chronic-cycling hypoxia in hypoxic tumor fractions.

## 4. Discussion

Adaptation processes of cancer cells to an adverse tumor microenvironment involve development of therapy resistance, which is represented in our experimental model of cancer cells selected by exposure to cycling severe hypoxia and reoxygenation stress. Beside selection-induced radioresistance [6,8,9,26], our previous work revealed that anoxia-tolerant cells also display resistance to chemotherapeutic agents like etoposide [34].

With our present work, we extend these findings with respect to chronic-cycling hypoxia-induced development of resistance to the MTH1-inhibitor TH1579. Of note, combination of TH1579 treatment with RT led to radiosensitization but was not able to counteract increased radioresistance induced by adaptation to chronic-cycling hypoxia/reoxygenation stress. However, importantly, disruption of redox homeostasis using PLN sensitized anoxia-tolerant cancer cells to MTH1 inhibition. Hence, application of a polymodal treatment using TH1579 and PLN was capable to overcome chronic-cycling hypoxia-induced radioresistance, under both normoxic and acute hypoxic treatment conditions. On a mechanistic level, our data revealed a glutathione-driven compensatory mechanism providing TH1579 resistance potentiated by acute and chronic-cycling hypoxia. Functionally, increased formation of ROS and oxidative DNA damage was observed upon multimodal treatment combining TH1579 and PLN in anoxia-tolerant cells when compared to oxic control cells disturbing important acquired mechanisms of anoxia-tolerant cells responsible for radioresitance.

MTH1 represents a promising target in different tumor entities [20,35,36]. However, various cellular processes could lead to MTH1-inhibitor resistance in the context of acute and chronic-cycling hypoxia. Others have described different resistance mechanisms to MTH1-inhibition, such as MTH1-independent 8-oxo-dGTPase activity [22] or dependence of TH1579 efficacy on tyrosine kinase AXL and caveolin-1 (CAV-1) [37]. Furthermore, reduced activity of BER system involving MTH1, OGG1 and FEN1 in hypoxic conditions [24] could lead to increased formation of observed oxidative DNA damage due to decreased repair capacity and thereby could render cancer cells less dependent to MTH1.

Instead, here we uncover a resistance mechanism towards MTH1-inhibition in form of increased antioxidant capacity as a consequence of microenvironmental or therapeutic stress.

In our previous work, upregulation of glutamic oxaloacetic transaminase 1 (GOT1) resulted in a glutamine-dependent increase of GSH [6]; moreover, increased activity of antioxidant-associated mitochondrial transport systems, namely SLC25A1 [9] and SLC25A10 [8] contributed to improved antioxidant defense in these cells by interference with cellular and mitochondrial GSH pools. Others have described increased NADPH production for GSH regeneration involving ubiqutin C-terminal hydrolase-L1 (UCHL1)/hypoxia-inducible factor 1 (HIF1) axis [10]. Additionally, cysteine-dependent novel synthesis of GSH as a consequence of ER stress downstream signaling of double-stranded RNA-activated protein kinase-like endoplasmic reticulum kinase (PERK)/eukaryotic initiation factor 2α (eIF2α)-dependent arm of the unfolded protein response was reported [7].

Instead, exposure to acute hypoxia led to increased HIF1-signalling and went along with activation of stress response cascades involving nuclear factor erythroid-2 like 2 (Nrf2) among others [38]. Nrf2 is considered as a master regulator of the cellular antioxidant defense driving chemo- and radioresistance [39] and is also induced upon RT-associated ROS-bursts [40].

Interestingly, exposure to acute hypoxia/reoxygenation stress and IR are both known to trigger formation of ROS [13] and both factors are counteracted by improved antioxidant defense. Thus, MTH1-inhibitor resistance in our hands is highly likely to be mediated by improved antioxidant defense in anoxia-tolerant cells. We speculate that functional sanitization of oxidized nucleotides only becomes crucial for survival of anoxia-tolerant cancer cells when redox homeostasis is impaired. Our data indicates enhanced ROS and oxidative DNA damage formation upon combined treatment of PLN and TH1579 than with the respective single treatments, especially in anoxia-tolerant cancer cells. This assumption is further supported by severely impaired short- and long-term cell survival upon combined treatment with PLN and TH1579. However, the molecular determinants of MTH1-inhibitor resistance need to be further investigated to design clinically useful treatment strategies.

Targeting the cellular antioxidant defense remains challenging because of the limited availability of specific and clinically useful drugs [41]. Due to the complex interconnections between energy metabolism and redox homeostasis in cancer [42], it might be possible to design rational treatment strategies targeting both factors concurrently. Previously, we and others have demonstrated preclinically that targeting of the mitochondrial carboxylate carriers SLC25A1 or SLC25A10 represent a strategy to target energy metabolism and redox homeostasis via one target at the same time [8,9,43,44].

In line with previous reports of others, we observe that increased oxidative stress sensitizes cancer cells to MTH1-inhibition [23]. Moreover, the present study further supports the established concept that targeting the antioxidant defense allows to increase ROS-dependent damage and is suited to improve the efficacy of chemo- or radiotherapy (RT).

Herein, indirect challenging of redox homeostasis by excessive induction of ROS represents a less complex and clinically relevant alternative, since RT but also several established and eligible chemotherapeutic agents are known to induce ROS [13,14]. Hence, for translation to the clinical setting our data suggest combining MTH1-inhibitors with established radio-chemotherapy or other approaches to impair redox homeostasis to counteract resistance mechanisms associated with improved antioxidant defense. In the future, it will be crucial to further investigate the mediating factors of MTH1-inhibitor resistance to potentially increase its antitumor efficacy. For clinical translation it will be also important to elucidate the mechanisms driving increased GSH in individual tumors to select appropriate combinatorial personalized treatment.

## 5. Conclusions

The present study reveals that the efficacy of MTH1-inhibition can be improved by targeting antioxidant defense in context of acute hypoxia but also after adaptation to chronic-cycling hypoxia in cancer cells of different tumor entities. In more detail, MTH1-inhibitor resistance was mediated by improved antioxidant capacity and could be disrupted by depleting cellular GSH. With respect to the clinical situation, our data suggest that combination of MTH1-Inhibition with ROS-inducing therapies like radio-chemotherapy could increase the therapeutic efficacy and counteract potential resistance mechanisms associated with improved redox balance.

## Data Availability

All data generated or analysed during this study are included in this published article and its Supplementary Materials files.

## Supplementary Materials

The following are available online at https://www.mdpi.com/article/10.3390/cells10113040/s1, Figure S1: Inhibition of MTH1 combined with inhibition of glutathione regeneration induces cell death without ionizing radiation (IR), Figure S2: Ionizing radiation (IR) or acute hypoxia triggers antioxidant defense which can be counteracted by inhibition of MTH1 and glutathione regeneration, Figure S3: Inhibition of MTH1 alone and combined with inhibition of glutathione regeneration leads to increased oxidative DNA damage, Figure S4: Inhibition of MTH1 combined with inhibition of glutathione regeneration reduces long-term survival without IR.

## References

[1] Dagogo-Jack I., Shaw A.T. (2018). Tumour heterogeneity and resistance to cancer therapies. Nat. Rev. Clin. Oncol..

[2] Hanahan D., Weinberg R.A. (2011). Hallmarks of Cancer: The next generation. Cell.

[3] Torre L.A., Siegel R.L., Ward E.M., Jemal A. (2016). Global cancer incidence and mortality rates and trends—An update. Cancer Epidemiol. Biomark. Prev..

[4] Harris A.L. (2002). Hypoxia—A key regulatory factor in tumour growth. Nat. Rev. Cancer.

[5] DeBerardinis R.J., Chandel N.S. (2016). Fundamentals of cancer metabolism. Sci. Adv..

[6] Matschke J., Riffkin H., Klein D., Handrick R., Lüdemann L., Metzen E., Shlomi T., Stuschke M., Jendrossek V. (2016). Targeted inhibition of glutamine-dependent glutathione metabolism overcomes death resistance induced by chronic cycling hypoxia. Antioxid. Redox Signal..

[7] Rouschop K.M., Dubois L.J., Keulers T.G., Beucken T.V.D., Lambin P., Bussink J., van der Kogel A.J., Koritzinsky M., Wouters B.G. (2013). PERK/eIF2 signaling protects therapy resistant hypoxic cells through induction of glutathione synthesis and protection against ROS. Proc. Natl. Acad. Sci. USA.

[8] Hlouschek J., Ritter V., Wirsdörfer F., Klein D., Jendrossek V., Matschke J. (2018). Targeting SLC25A10 alleviates improved antioxidant capacity and associated radioresistance of cancer cells induced by chronic-cycling hypoxia. Cancer Lett..

[9] Hlouschek J., Hansel C., Jendrossek V., Matschke J. (2018). The Mitochondrial Citrate Carrier (SLC25A1) sustains redox homeostasis and mitochondrial metabolism supporting radioresistance of cancer cells with tolerance to cycling severe hypoxia. Front. Oncol..

[10] Nakashima R., Goto Y., Koyasu S., Kobayashi M., Morinibu A., Yoshimura M., Hiraoka M., Hammond E.M., Harada H. (2017). UCHL1-HIF-1 axis-mediated antioxidant property of cancer cells as a therapeutic target for radiosensitization. Sci. Rep..

[11] Jiang H., Wang H., De Ridder M. (2018). Targeting antioxidant enzymes as a radiosensitizing strategy. Cancer Lett..

[12] Cairns R.A., Harris I., Mak T.W. (2011). Regulation of cancer cell metabolism. Nat. Rev. Cancer.

[13] Wang H., Jiang H., Van De Gucht M., De Ridder M. (2019). Hypoxic radioresistance: Can ROS be the key to overcome it?. Cancers.

[14] Yang H., Villani R.M., Wang H., Simpson M.J., Roberts M.S., Tang M., Liang X. (2018). The role of cellular reactive oxygen species in cancer chemotherapy. J. Exp. Clin. Cancer Res..

[15] Lieber M. (1997). The FEN-1 family of structure-specific nucleases in eukaryotic DNA replication, recombination and repair. BioEssays.

[16] Boiteux S., Radicella J.P. (2000). The human OGG1 Gene: Structure, functions, and its implication in the process of carcinogenesis. Arch. Biochem. Biophys..

[17] Powell S.N., Bindra R.S. (2009). Targeting the DNA damage response for cancer therapy. DNA Repair.

[18] Ashworth A., Lord C.J. (2018). Synthetic lethal therapies for cancer: What’s next after PARP inhibitors?. Nat. Rev. Clin. Oncol..

[19] Yap T.A., Plummer R., Azad N.S., Helleday T. (2019). The DNA damaging revolution: PARP inhibitors and beyond. Am. Soc. Clin. Oncol. Educ. Book.

[20] Gad H., Koolmeister T., Jemth A.-S., Eshtad S., Jacques S.A., Ström C.E., Svensson L.M., Schultz N., Lundbäck T., Einarsdottir B. (2014). MTH1 inhibition eradicates cancer by preventing sanitation of the dNTP pool. Nature.

[21] Kettle J.G., Alwan H., Bista M., Breed J., Davies N.L., Eckersley K., Fillery S., Foote K.M., Goodwin L., Jones D.R. (2016). Potent and selective inhibitors of MTH1 probe its role in cancer cell survival. J. Med. Chem..

[22] Samaranayake G.J., Troccoli C.I., Zhang L., Huynh M., Jayaraj C.J., Ji D., McPherson L., Onishi Y., Nguyen D.M., Robbins D.J. (2019). The existence of MTH1-independent 8-oxodGTPase activity in cancer cells as a compensatory mechanism against on-target effects of MTH1 inhibitors. Mol. Cancer Ther..

[23] Bräutigam L., Pudelko L., Jemth A.-S., Gad H., Narwal M., Gustafsson R., Karsten S., Puigvert J.C., Homan E., Berndt C. (2016). Hypoxic signaling and the cellular redox tumor environment determine sensitivity to MTH1 inhibition. Cancer Res..

[24] Kaplan A., Glazer P.M. (2019). Impact of hypoxia on DNA repair and genome integrity. Mutagenesis.

[25] Bristow R.G., Hill R.P. (2008). Hypoxia and metabolism. Hypoxia, DNA repair and genetic instability. Nat. Rev. Cancer.

[26] Matschke J., Wiebeck E., Hurst S., Rudner J., Jendrossek V. (2016). Role of SGK1 for fatty acid uptake, cell survival and radioresistance of NCI-H460 lung cancer cells exposed to acute or chronic cycling severe hypoxia. Radiat. Oncol..

[27] Bezerra D.P., Pessoa C., de Moraes M.O., Saker-Neto N., Silveira E.R., Costa-Lotufo L.V. (2013). Overview of the therapeutic potential of piplartine (piperlongumine). Eur. J. Pharm. Sci..

[28] Berglund U.W., Sanjiv K., Gad H., Kalderén C., Koolmeister T., Pham T., Gokturk C., Jafari R., Maddalo G., Seashore-Ludlow B. (2016). Validation and development of MTH1 inhibitors for treatment of cancer. Ann. Oncol..

[29] Caradec J., Sirab N., Keumeugni C., Moutereau S., Chimingqi M., Matar C., Revaud D., Bah M., Manivet P., Conti M. (2010). ‘Desperate house genes’: The dramatic example of hypoxia. Br. J. Cancer.

[30] Pfaffl M.W. (2001). A new mathematical model for relative quantification in real-time RT-PCR. Nucleic Acids Res..

[31] Thomas J.P., Lautermann J., Liedert B., Seiler F., Thomale J. (2006). High accumulation of platinum-DNA adducts in strial marginal cells of the cochlea is an early event in cisplatin but not carboplatin ototoxicity. Mol. Pharmacol..

[32] Franken N.A.P., Rodermond H.M., Stap J., Haveman J., Van Bree C. (2006). Clonogenic assay of cells in vitro. Nat. Protoc..

[33] Raj L., Ide T., Gurkar A.U., Foley M., Schenone M., Li X., Tolliday N.J., Golub T.R., Carr S.A., Shamji A.F. (2011). Selective killing of cancer cells by a small molecule targeting the stress response to ROS. Nature.

[34] Weinmann M., Jendrossek V., Güner D., Goecke B., Belka C. (2004). Cyclic exposure to hypoxia and reoxygenation selects for tumor cells with defects in mitochondrial apoptotic pathways. FASEB J..

[35] Pompsch M., Vogel J., Classen F., Kranz P., Iliakis G., Riffkin H., Brockmeier U., Metzen E. (2018). The presumed MTH1-inhibitor TH588 sensitizes colorectal carcinoma cells to ionizing radiation in hypoxia. BMC Cancer.

[36] McPherson L.A., Troccoli C.I., Ji D., Bowles A.E., Gardiner M.L., Mohsen M.G., Nagathihalli N.S., Nguyen D.M., Robbins D.J., Merchant N.B. (2019). Increased MTH1-specific 8-oxodGTPase activity is a hallmark of cancer in colon, lung and pancreatic tissue. DNA Repair.

[37] Das I., Gad H., Bräutigam L., Pudelko L., Tuominen R., Höiom V., Almlöf I., Rajagopal V., Hansson J., Helleday T. (2020). AXL and CAV-1 play a role for MTH1 inhibitor TH1579 sensitivity in cutaneous malignant melanoma. Cell Death Differ..

[38] Toth R.K., Warfel N.A. (2017). Strange Bedfellows: Nuclear Factor, Erythroid 2-Like 2 (Nrf2) and Hypoxia-Inducible Factor 1 (HIF-1) in Tumor Hypoxia. Antioxidants.

[39] Leinonen H.M., Kansanen E., Pölönen P., Heinäniemi M., Levonen A.-L. (2014). Role of the Keap1–Nrf2 Pathway in Cancer. Adv. Cancer Res..

[40] Sekhar K.R., Freeman M.L. (2015). Nrf2 promotes survival following exposure to ionizing radiation. Free. Radic. Biol. Med..

[41] Hatem E., El Banna N., Huang M.-E. (2017). Multifaceted roles of glutathione and glutathione-based systems in carcinogenesis and anticancer drug resistance. Antioxid. Redox Signal..

[42] De Santis M.C., Porporato P.E., Martini M., Morandi A. (2018). Signaling pathways regulating redox balance in cancer metabolism. Front. Oncol..

[43] Fernandez H.R., Gadre S.M., Tan M., Graham G.T., Mosaoa R., Ongkeko M.S., Kim K.A., Riggins R.B., Parasido E., Petrini I. (2018). The mitochondrial citrate carrier, SLC25A1, drives stemness and therapy resistance in non-small cell lung cancer. Cell Death Differ..

[44] Zhao Q., Zhou X., Curbo S., Karlsson A. (2018). Metformin downregulates the mitochondrial carrier SLC25A10 in a glucose dependent manner. Biochem. Pharmacol..

