# Supplementary material for: Adaptation to Chronic-Cycling Hypoxia Renders Cancer Cells Resistant to MTH1-Inhibitor Treatment Which Can Be Counteracted by Glutathione Depletion
Source: Cells. 2021 Nov 5;10(11):3040. doi: 10.3390/cells10113040 (PMC8616547; doi:10.3390/cells10113040)
Supplement: Supplementary file 1 [file cells-10-03040-s001.zip › cells-1428897-supplementary.pdf]

cells MDPI

*Supplementary Materials*

# Adaptation to Chronic-Cycling Hypoxia Renders Cancer Cells Resistant to MTH1-Inhibitor Treatment Which Can Be Counteracted by Glutathione Depletion

**Christine Hansel [1,†], Julian Hlouschek [1,†], Kexu Xiang [1], Margarita Melnikova [1], Juergen Thomale [1], Thomas Helleday [2], Verena Jendrossek [1] and Johann Matschke [1,*]**

1 Institute of Cell Biology (Cancer Research), University Hospital Essen, University of Duisburg-Essen, 45147 Essen, Germany; Christine.Hansel@uk-essen.de (C.H.); julian.hlouschek@stud.uni-due.de (J.H.); kexu.xiang@uk-essen.de (K.X.); margarita.melnikova@uk-essen.de (M.M.); Juergen.Thomale@uk-essen.de (J.T.); verena.jendrossek@uni-due.de (V.J.)
2 Science for Life Laboratory, Karolinska Institutet, 17121 Stockholm, Sweden; thomas.helleday@scilifelab.se
* Correspondence: johann.matschke@uk-essen.de; Tel.: +49-201-7234234; Fax: +49-201-7235904
† These authors contributed equally.

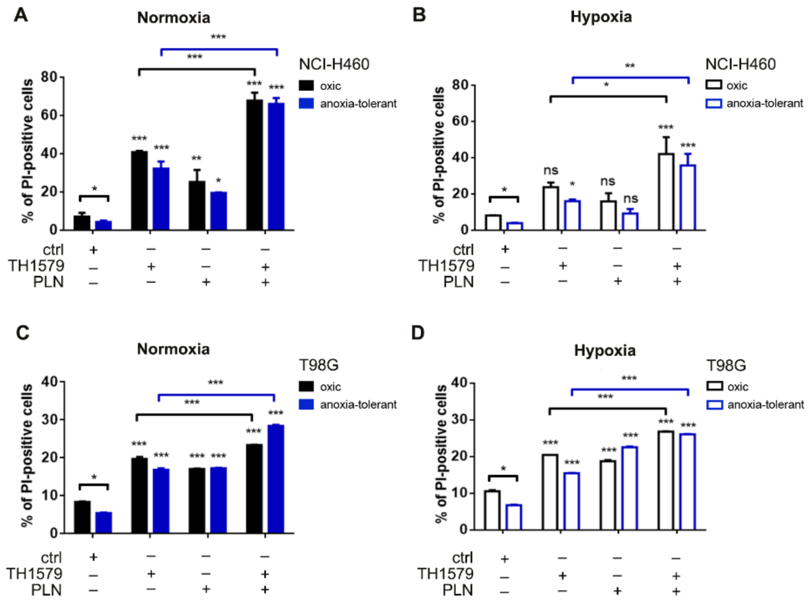


E

| | | NCI-H460 oxic | | | | NCI-H460 anoxia-tolerant | | | |
|---|---|---|---|---|---|---|---|---|---|
| | | Nx | | Hx | | Nx | | Hx | |
| | | Mean | SEM | Mean | SEM | Mean | SEM | Mean | SEM |
| 0Gy | ctrl | 7,19 | 1,94 | 8,07 | 0,17 | 4,37 | 0,67 | 3,92 | 0,16 |
| | TH1579 | 40,80 | 0,61 | 23,68 | 2,62 | 32,22 | 3,76 | 16,05 | 0,99 |
| | PLN | 25,27 | 6,24 | 15,91 | 4,51 | 19,50 | 0,30 | 9,21 | 2,50 |
| | TH1579 + PLN | 67,70 | 4,20 | 42,00 | 9,30 | 66,00 | 3,10 | 35,77 | 6,40 |
| 10Gy | ctrl | 22,61 | 1,87 | 25,64 | 2,70 | 12,24 | 1,62 | 11,96 | 1,27 |
| | TH1579 | 34,15 | 4,44 | 23,77 | 3,79 | 20,07 | 0,87 | 13,17 | 2,67 |
| | PLN | 43,20 | 5,90 | 18,05 | 4,95 | 49,45 | 1,45 | 18,67 | 0,69 |
| | TH1579 + PLN | 69,35 | 3,55 | 36,53 | 8,82 | 69,90 | 1,60 | 37,17 | 5,50 |

| | | T98G oxic | | | | T98G anoxia-tolerant | | | |
|---|---|---|---|---|---|---|---|---|---|
| | | Nx | | Hx | | Nx | | Hx | |
| | | Mean | SEM | Mean | SEM | Mean | SEM | Mean | SEM |
| 0Gy | ctrl | 8,39 | 0,14 | 10,50 | 0,30 | 5,50 | 0,09 | 6,73 | 0,12 |
| | TH1579 | 19,73 | 0,55 | 20,40 | 0,00 | 16,85 | 0,45 | 15,40 | 0,10 |
| | PLN | 17,07 | 0,12 | 18,70 | 0,30 | 17,30 | 0,10 | 22,50 | 0,20 |
| | TH1579 + PLN | 23,33 | 0,12 | 26,75 | 0,05 | 28,40 | 0,30 | 26,00 | 0,10 |
| 10Gy | ctrl | 19,75 | 0,05 | 13,35 | 0,05 | 13,70 | 0,10 | 10,95 | 0,05 |
| | TH1579 | 37,30 | 0,40 | 26,80 | 0,60 | 25,30 | 0,10 | 22,25 | 0,65 |
| | PLN | 26,35 | 0,15 | 22,10 | 0,30 | 30,55 | 0,25 | 36,60 | 0,40 |
| | TH1579 + PLN | 37,10 | 0,80 | 28,55 | 0,25 | 41,15 | 0,05 | 41,00 | 0,40 |

**Figure S1.** Inhibition of MTH1 combined with inhibition of glutathione regeneration induces cell death without ionizing radiation (IR). Impact of MTH1 inhibition by TH1579 (1 µM) alone and in

combination with GSH-Inhibitor PLN (10 µM) on cell death under normoxic (21% $O_2$) and hypoxic (0.2% $O_2$) conditions was examined. **A)** Fraction of dead (Propidium iodide (PI)-positive) NCI-H460 cells 72 h after respective inhibitor treatments under normoxia determined by flow cytometry. **B)** Fraction of dead (PI-positive) NCI-H460 cells 72 h after respective inhibitor treatments under hypoxia determined by flow cytometry. **C)** Fraction of dead (PI-positive) T98G cells 72 h after respective inhibitor treatments under normoxia determined by flow cytometry. **D)** Fraction of dead (PI-positive) T98G cells 72 h after respective inhibitor treatments under hypoxia determined by flow cytometry. **E)** Measured mean % values for the PI-positive cells with respective SEM-values. Mean values ± SEM are shown, n=3 (* p≤0.05, ** p≤0.01, *** p≤0.001; 2-way ANOVA with Tukey post-test).

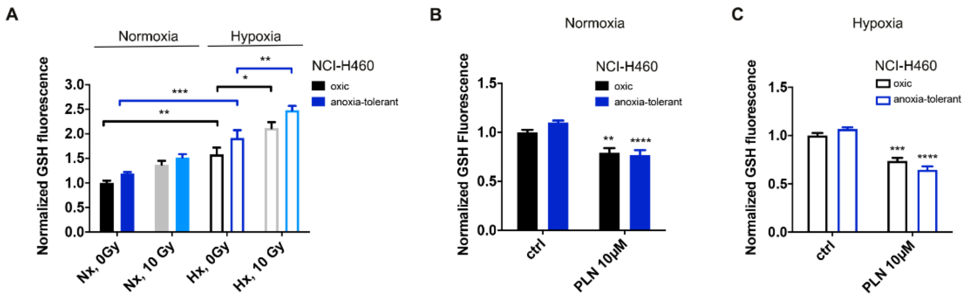


**Figure S2.** Ionizing radiation (IR) or acute hypoxia triggers antioxidant defense which can be counteracted by inhibition of MTH1 and glutathione regeneration**.** Impact of IR and/or acute hypoxia on antioxidant capacity of oxic and anoxia-tolerant cells and the potential of piperlongumine (PLN) and MTH1-inhibitor TH1579 are shown. **A)** Relative fluorescence of reduced glutathione (GSH) normalized to cell number in NCI-H460 oxic and anoxia-tolerant cells 24 h after exposure to 10 Gy IR, acute hypoxia or both. **B-C)** Relative levels of GSH after 2 h of PLN-treatment under normoxic (B) and hypoxic (C) conditions. Bar graph indicates GSH levels relative to untreated oxic control in NCI-H460 oxic and anoxia-tolerant cells. Mean values ± SEM are shown, n=3 (ns: not significant, * p≤0.05, ** p≤0.01, *** p≤0.001, **** p≤0.0001; 2-way ANOVA with Tukey post-test).

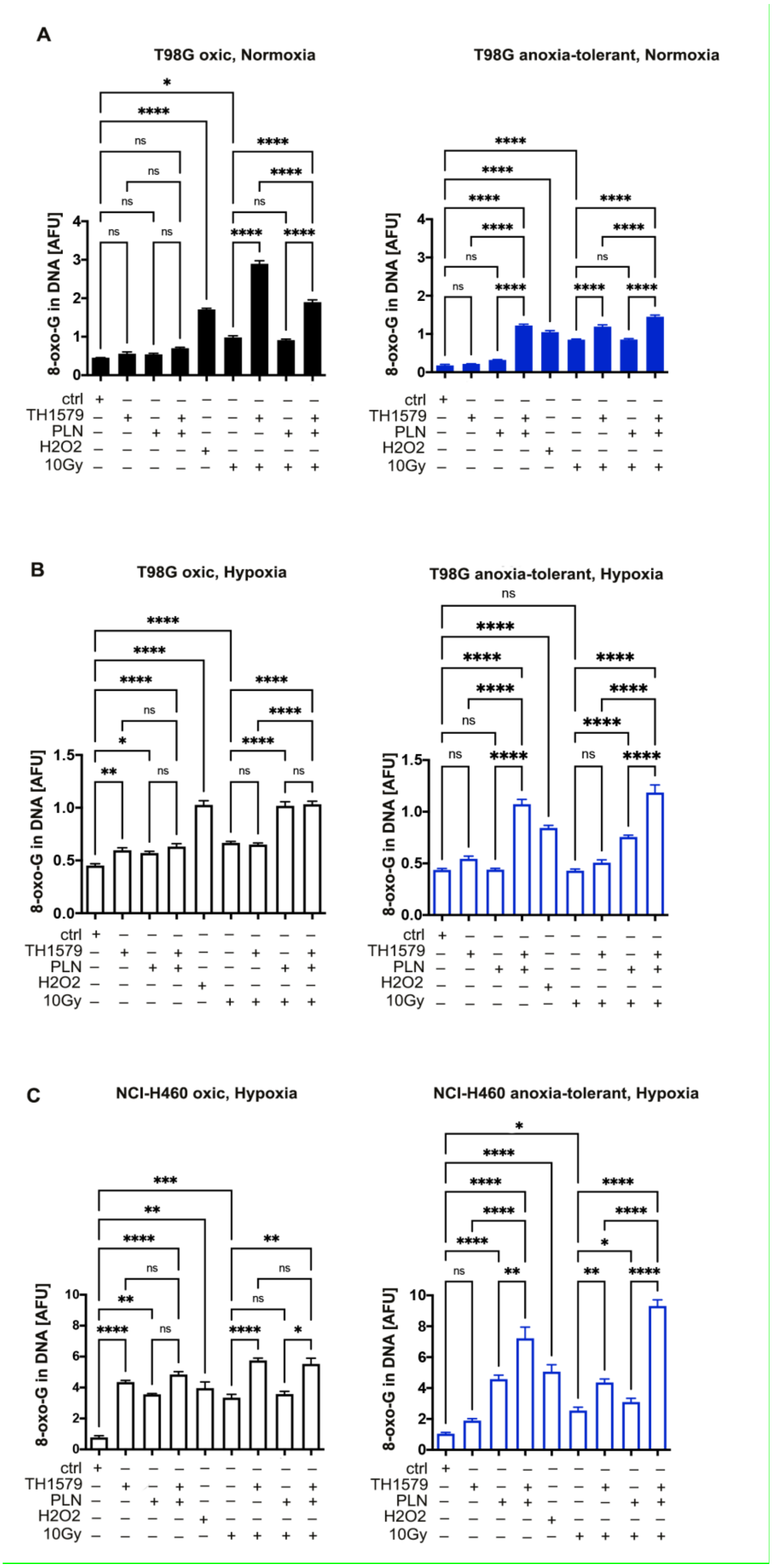


**Figure S3.** Inhibition of MTH1 alone and combined with inhibition of glutathione regeneration leads to increased oxidative DNA damage. Effect of IR and/or acute hypoxia on formation of oxidative DNA damage in oxic and anoxia-tolerant T98G or NCI-H460 cells and the alterations upon

24 h of treatment with PLN and TH1579 were investigated. **A)** Bar graphs indicating arbitrary fluorescence units (AFU) of 8-oxo-G of oxic (left panel) and anoxia-tolerant (right panel) T98G cells in nucleus area after treatment under normoxic conditions. **B)** AFU of 8-oxo-G of oxic (left panel) and anoxia-tolerant (right panel) T98G cells in nucleus area after treatment under hypoxic conditions. **C)** AFU of 8-oxo-G of oxic (left panel) and anoxia-tolerant (right panel) NCI-H460 cells in nucleus area after treatment under hypoxic conditions. Mean values ± SEM are shown, n=3 (ns: not significant, * p≤0.05, ** p≤0.01, *** p≤0.001, **** p≤0.0001; 2-way ANOVA with Tukey post-test).

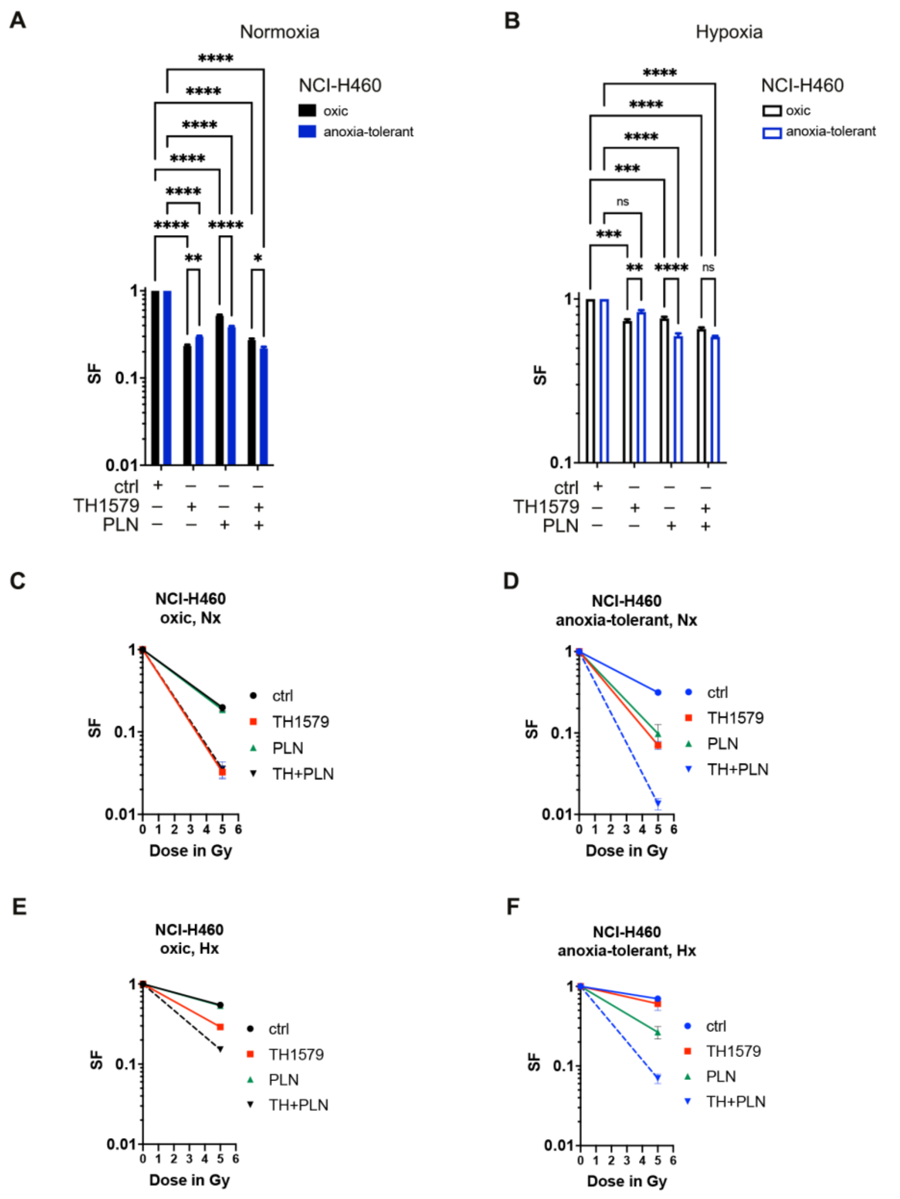


**Figure S4.** Inhibition of MTH1 combined with inhibition of glutathione regeneration reduces long-term survival without IR. Impact of MTH1 inhibition by TH1579 (1 µM) alone and in combination with GSH-Inhibitor PLN (10 µM) on long-term survival under normoxic (21% O2) and hypoxic (0.2% O2) conditions was examined. **A)** Survival fraction (SF) of clonogenic survival of NCI-H460 oxic and anoxia-tolerant cells upon respective drug treatments without IR under normoxic conditions is presented in a bar graph. **B)** Survival fraction (SF) of clonogenic survival of NCI-H460 oxic and anoxia-tolerant cells upon respective drug treatments without IR under hypoxic conditions is presented in a bar graph. **C-F)** Survival curves of NCI-H460 oxic (C) or NCI-H460 anoxia-tolerant (D) cancer cells treated in Nx or Hx (E, F) representing the mean survival fraction (SF) ± SEM of clonogenic survival upon IR alone or in combination with drug-treatment as indicated (n=3). Mean

values ± SEM are shown, n=3 (ns: not significant, * p≤0.05, ** p≤0.01, *** p≤0.001, **** p≤0.0001; 2-way ANOVA with Tukey post-test)
